# Optimizing Nasal Potential Difference Analysis for CFTR Modulator Development: Assessment of Ivacaftor in CF Subjects with the *G551D-CFTR* Mutation

**DOI:** 10.1371/journal.pone.0066955

**Published:** 2013-07-26

**Authors:** Steven M. Rowe, Bo Liu, Aubrey Hill, Heather Hathorne, Morty Cohen, John R. Beamer, Frank J. Accurso, Qunming Dong, Claudia L. Ordoñez, Anne J. Stone, Eric R. Olson, John P. Clancy, for the VX06-770-101 Study Group

**Affiliations:** 1 University of Alabama at Birmingham, Birmingham, Alabama, United States of America; 2 Seattle Children's Hospital, Seattle, Washington, United States of America; 3 University of Colorado Denver, Aurora, Colorado, United States of America; 4 Vertex Pharmaceuticals Incorporated, Cambridge, Massachusetts, United States of America; 5 Cincinnati Children's Hospital Medical Center and the University of Cincinnati, Cincinnati, Ohio, United States of America; The Ohio State University, United States of America

## Abstract

Nasal potential difference (NPD) is used as a biomarker of the cystic fibrosis transmembrane conductance regulator (CFTR) and epithelial sodium channel (ENaC) activity. We evaluated methods to detect changes in chloride and sodium transport by NPD based on a secondary analysis of a Phase II CFTR-modulator study. Thirty-nine subjects with CF who also had the *G551D-CFTR* mutation were randomized to receive ivacaftor (Kalydeco™; also known as VX-770) in four doses or placebo twice daily for at least 14 days. All data were analyzed by a single investigator who was blinded to treatment assignment. We compared three analysis methods to determine the best approach to quantify changes in chloride and sodium transport: (1) the average of both nostrils; (2) the most-polarized nostril at each visit; and (3) the most-polarized nostril at screening carried forward. Parameters of ion transport included the PD change with zero chloride plus isoproterenol (CFTR activity), the basal PD, Ringer's PD, and change in PD with amiloride (measurements of ENaC activity), and the delta NPD (measuring CFTR and ENaC activity). The average and most-polarized nostril at each visit were most sensitive to changes in chloride and sodium transport, whereas the most-polarized nostril at screening carried forward was less discriminatory. Based on our findings, NPD studies should assess both nostrils rather than a single nostril. We also found that changes in CFTR activity were more readily detected than changes in ENaC activity, and that rigorous standardization was associated with relatively good within-subject reproducibility in placebo-treated subjects (±2.8 mV). Therefore, we have confirmed an assay of reasonable reproducibility for detecting chloride-transport improvements in response to CFTR modulation.

## Introduction

Cystic fibrosis (CF) is a common genetic disorder resulting from mutations in the cystic fibrosis transmembrane conductance regulator (*CFTR*) gene [1]. In the airways, the CFTR protein channel transports chloride (Cl^−^) and bicarbonate and regulates other ion-transport pathways including alternate Cl^−^ channels and the epithelial sodium (Na^+^) channel (ENaC) [2]–[5]. CFTR dysfunction interrupts the normal regulation of salt and water transport leading to desiccated airway secretions, mucus stasis, infection, and inflammation, which culminate in bronchiectasis and respiratory failure.

CFTR activity can be measured *in vivo* using nasal potential difference (NPD) and sweat Cl^−^ analysis [6]. NPD is unique because it isolates both ENaC and CFTR Cl^−^ channel function independent of other ion transport processes, providing a real-time estimate of CFTR and ENaC activity. NPD measurements correlate with the CF clinical phenotype in retrospective studies [7], [8] and are frequently used in clinical trials to detect rescue of CFTR activity in humans [8]–[22]. However, due to lack of consensus regarding optimal methodology, it remains unclear as to what methods are superior [23], [24].

Ivacaftor (Kalydeco™; also known as VX-770) is a CFTR potentiator approved for the treatment of CF caused by the *G551D* mutation (in patients ≥6 years of age) by increasing gating and anion channel activity of G551D-CFTR (found in approximately 4% of patients [25]). A Phase II clinical trial of ivacaftor demonstrated dose-dependent improvements in CFTR biomarkers (including NPD and sweat Cl^−^ along with clinical efficacy measures) in subjects with the *G551D-CFTR* mutation [22]. This was the first published study employing the latest NPD standardization across centers in the United States, and provided a unique opportunity to optimize NPD parameters to detect modulator activity. In this secondary analysis of data from the Phase II trial, we evaluated three methods for determining NPD and the capacity of each to detect changes in various NPD parameters.

## Methods

The details of the clinical trial have been previously published [22]. This study was performed at 12 participating sites across the United States. Institutional review board (IRB) approval at each participating center and written informed consent were obtained for all enrolled study subjects for a two-part, randomized, placebo-controlled trial examining different doses of ivacaftor in subjects with the *G551D-CFTR* mutation on at least one allele. The NCT number is NCT00457821, and the study title is ‘A Phase 2a, Randomized, Double-Blind, Placebo-Controlled Study of VX-770 to Evaluate Safety, Pharmacokinetics, and Biomarkers of CFTR Activity in Cystic Fibrosis (CF) Subjects with Genotype G551D’, URL http://clinicaltrials.gov/ct2/show/NCT00457821.

Subjects were enrolled based on a diagnosis of CF, age >18 years, lung function >40% of the predicted value for age, sex, and height, and genotype for the *G511D-CFTR* mutation. Since >90% of CF patients are White, the vast majority of enrolled study subjects were White. The study had insufficient numbers of subjects for conclusions to be made regarding gender effects, but gender is included for descriptive purposes.

Subjects were randomized to receive placebo (n = 12) or ivacaftor in doses of 25 mg (n = 8), 75 mg (n = 16), 150 mg (n = 16), or 250 mg (n = 7) that were administered orally every 12 hours for 14–28 days. The analysis in this report combined Day 14 values from Parts 1 and 2 of the study. In addition, similar NPD data (difference between Day 1 and Day 14 values) from placebo subjects in a subsequent Phase II study of the putative *F508del-CFTR* corrector lumacaftor (also known as VX-809) (n = 13 subjects, all *F508del-CFTR* homozygous) were included in aggregate analysis [26]. IRB approval at each participating center and written informed consent were obtained for all enrolled study subjects in the lumacaftor trial.

Several NPD parameters were assessed (difference from pretreatment values through Day 14) including: (1) zero Cl^−^ plus isoproterenol (a direct measure of CFTR activity); (2) average basal PD (five sites in the inferior meatus), (3) maximal basal PD, (4) Ringer's basal PD, (5) change in PD with amiloride, and (6) percent change in PD with amiloride [(2) through (6) are all measurements of Na^+^ transport); and (7) the delta NPD (the change in PD from end of Ringer's perfusion to end of zero Cl^−^ plus isoproterenol perfusion – a combined measure of both CFTR and ENaC activity).

Three NPD methods were compared: (1) the average of both nostrils; (2) the most-polarized nostril at each visit; and (3) the most-polarized nostril at screening carried forward for the subsequent measurements. NPD analysis and statistical analysis methods are included in Methods S1 in File S1.

## Results

### Study Subject Demographic Information, Safety, and Tolerability

The baseline characteristics of study subjects have been summarized in a separate publication [22] and are shown in Table 1. The safety and tolerability profile of ivacaftor in this study also have been described previously [22].

**Table 1:** Baseline characteristics of study participants.

Characteristic	Part 1						Part 2			
	Placebo (n = 4)	Ivacaftor				TOTAL (n = 20)	Placebo (n = 4)	Ivacaftor		TOTAL (n = 19)
		25 mg/75 mg (n = 4)	75 mg/25 mg (n = 4)	75 mg/150 mg (n = 4)	150 mg/75 mg (n = 4)			150 mg (n = 8)	250 mg (n = 7)	
**Sex**, n (%)										
Male	2 (50)	1 (25)	4 (100)	1 (25)	1 (25)	9 (45)	3 (75)	3 (38)	4 (57)	10 (53)
Female	2 (50)	3 (75)	0	3 (75)	3 (75)	11 (55)	1 (25)	5 (63)	3 (43)	9 (47)
**Race**, n (%)										
White	4 (100)	4 (100)	4 (100)	4 (100)	4 (100)	20 (100)	4 (100)	8 (100)	7 (100)	19 (100)
**Age**, yr, median (range)	36 (19–48)	31 (22–51)	41 (22–50)	26 (19–34)	21 (19–33)	30 (19–51)	24 (18–42)	23 (18–40)	21 (20–38)	21 (18–42)
**BMI**, kg/m^2^, median (range)	23 (22–29)	23 (20–24)	24 (19–27)	20 (19–24)	21 (17–26)	23 (17–29)	22 (21–23)	22 (20–23)	23 (20–25)	22 (20–25)
***CFTR*** ** genotype**										
G551D/F508del	3 (75)	4 (100)	4 (100)	2 (50)	3 (75)	16 (80)	4 (100)	7 (88)	5 (71)	16 (84)
G551D/1078delT	1 (25)	–	–	–	–	1 (5)	–	–	–	–
G551D/G551D	–	–	–	–	1 (25)	1 (5)	–	–	–	–
G551D/N1303K	–	–	–	1 (25)	–	1 (5)	–	–	–	–
G551D/R553X	–	–	–	1 (25)	–	1 (5)	–	–	–	–
G551D/3849+10 kbC	–	–	–	–	–	–	–	–	1 (14)	1 (5)
G551D/6214→1G+7T	–	–	–	–	–	–	–	1 (13)	–	1 (5)
G551D/G542X	–	–	–	–	–	–	–	–	1 (14)	1 (5)
**% Predicted FEV_1_**, median (range)										
40% to <70%, n (%)	3 (75)	3 (75)	4 (100)	2 (50)	4 (100)	16 (80)	2 (50)	5 (63)	3 (43)	10 (53)
70% to <90%, n (%)	–	–	–	1 (25)	–	1 (5)				
>90%, n (%)	–	1 (25)	–	1 (25)	–	3 (15)	2 (50)	1 (13)	1 (14)	4 (21)
**Sweat chloride**, mmol/L, median (range)	105.25 (97.00–112.00)	107.50 (60.00– 117.00)	104.50 (102.00–117.00)	97.50 (92.00–102.50)	99.00 (83.00–104.00)	102.00 (60.00–117.00)	93.75 (88.00–109.50)	100.13 (86.75–112.50)	97.25 (84.75–115.75)	95.50 (84.75–115.75)

BMI, body-mass index; CFTR, cystic fibrosis transmembrane conductance regulator; FEV_1_, forced expiratory volume in one second.

### Stability of NPD Solutions

Stability of NPD solutions is summarized in Table S1 in File S1. The concentrations of Na^+^, Cl^−^, amiloride, and pH remained within 10% of initial values over 12 months under refrigerated (2°–8°C) conditions.

### Changes in CFTR-Dependent Cl^−^ Transport

**Figure 1:**
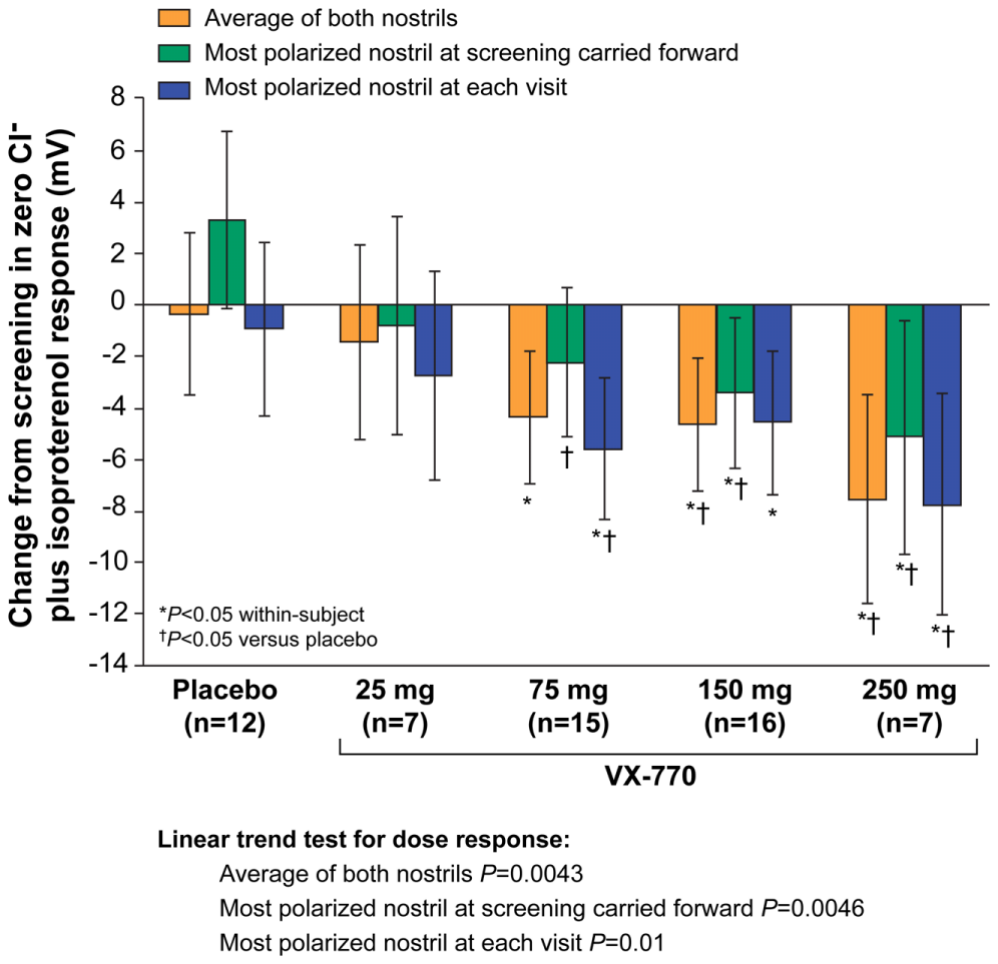
Change from screening to Day 14 in CFTR activity (zero Cl^−^ plus isoproterenol response), Parts 1 and 2 combined. Data are shown for the analysis of the average of both nostrils, the most-polarized nostril at screening carried forward, and the most-polarized nostril at each visit. The dose-dependent linear trends were statistically significant for all three analyses (*P*<0.02).

We applied the three NPD analysis methods to examine the change in zero Cl^−^ plus isoproterenol, the principle measure of CFTR-dependent Cl^−^ activity (Figure 1). Significant improvements in Cl^−^ activity were observed at the 75-mg, 150-mg, and 250-mg dose groups for all three methods, including both within-subject and placebo comparisons. The magnitude of change in Cl^−^ activity was smaller with the most-polarized nostril at screening carried forward compared to the other two analysis methods (without a reduction in variance), suggesting this method was inferior for detecting a change in PD produced by an efficacious CFTR modulator. Each method exhibited a significant linear test for trend (*P*≤0.01).

### Changes in Na^+^ Transport

**Figure 2:**
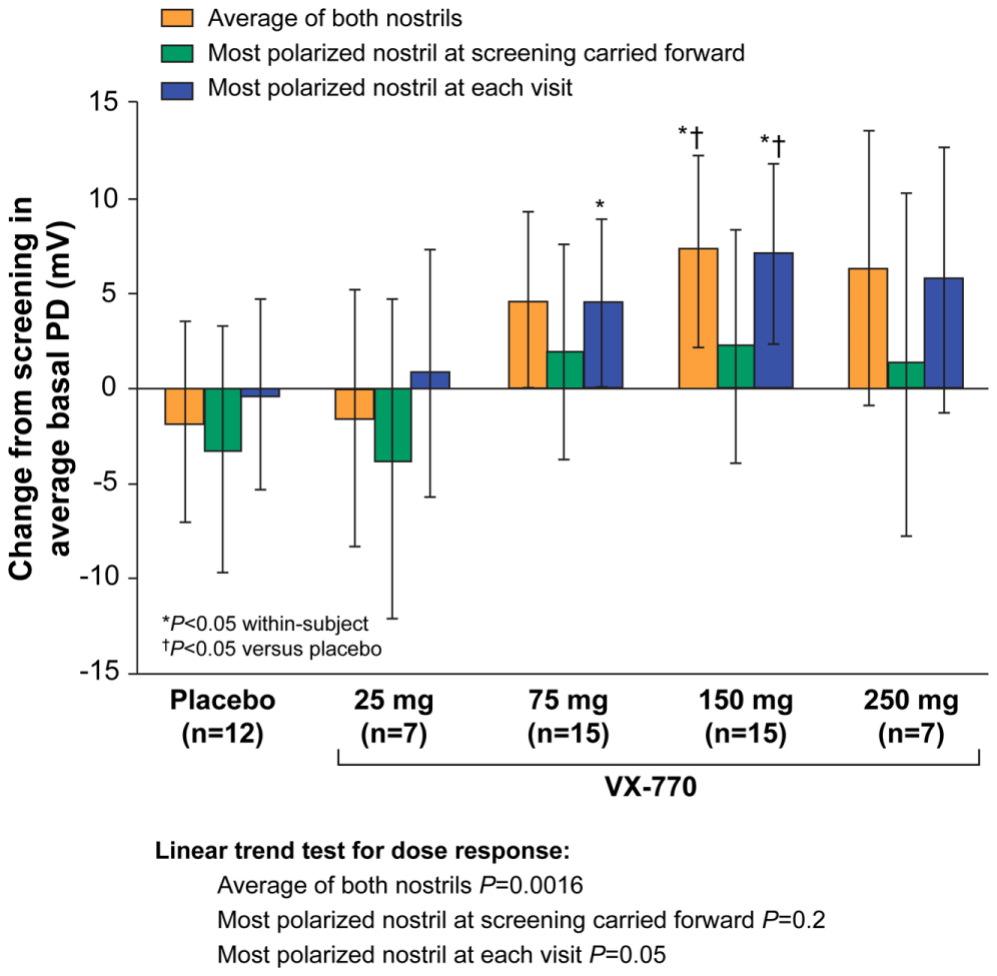
Change from screening to Day 14 in ENaC activity (average basal PD, Parts 1 and 2 combined). Data are shown for the analysis of the average of both nostrils, the most-polarized nostril at screening carried forward, and the most-polarized nostril at each visit. Significant dose-dependent linear trends were demonstrated for the average of both nostrils and the most-polarized nostril at each visit (*P*<0.02).

Figure 2 summarizes the change in average basal PD for the three analysis methods. Both the average of both nostrils and the most-polarized nostril at each visit methods demonstrated dose-dependent improvements in baseline hyperpolarization with treatment. In contrast, the most-polarized nostril at screening carried forward analysis demonstrated only minimal improvement with ivacaftor treatment, and this analysis method had the widest confidence interval among patients treated with placebo. As opposed to Cl^−^ transport, the treatment effect for Na^+^ transport was not significant at the 250-mg dose.

**Figure 3:**
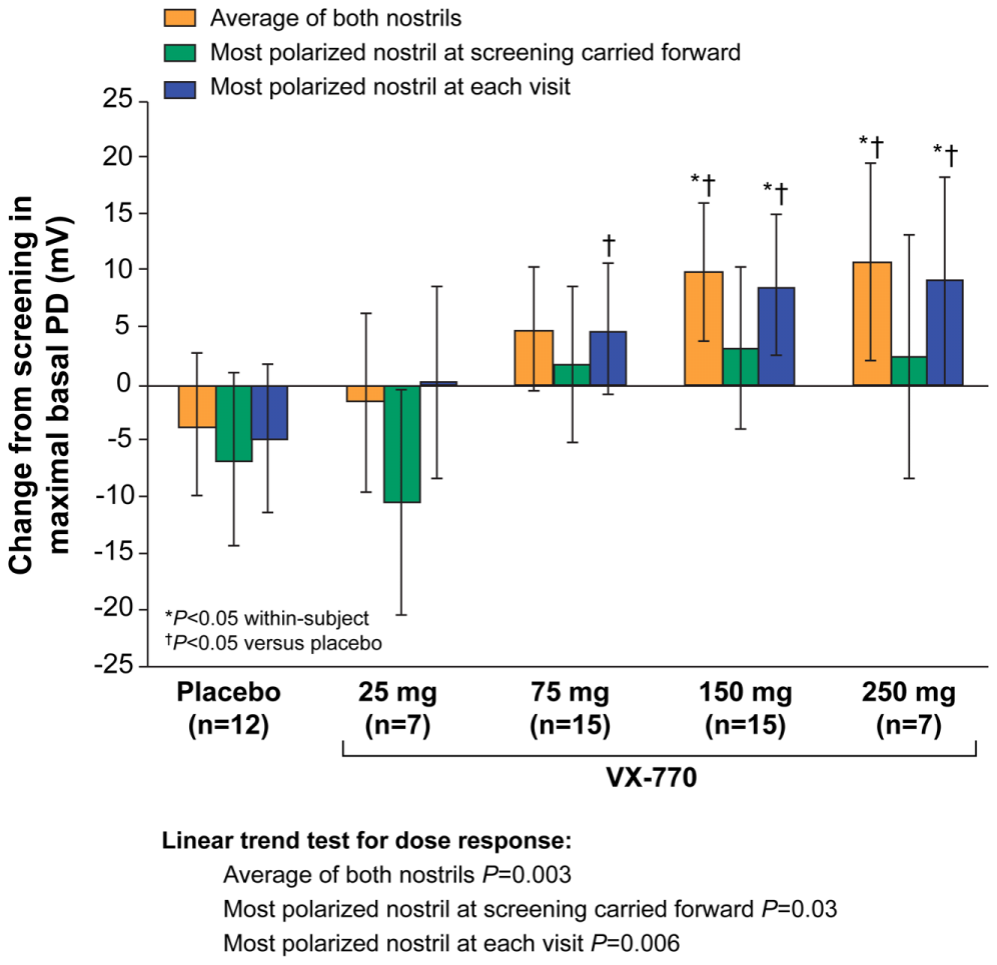
Change from screening to Day 14 in ENaC activity (maximum basal PD, Parts 1 and 2 combined). Data are shown for the analysis of the average of both nostrils, the most-polarized nostril at screening carried forward, and the most-polarized nostril at each visit. The dose-dependent linear trends were statistically significant for all three analyses (*P*<0.05).

Figure 3 shows the change in maximal basal PD for the three analysis methods. Significant, dose-dependent reductions in hyperpolarization were noted for the average of both nostrils and the most-polarized nostril at each visit, which extended to the 150-mg and 250-mg dose groups, compared with placebo. In contrast, the most-polarized nostril at screening carried forward did not demonstrate significant changes at 75, 150, or 250 mg (within-subject or vs placebo). The change in Ringer's PD for each analytic method exhibited similar findings to that seen with the average basal PD and the maximal basal PD parameters (Figure S1 in File S1). Only the average of both nostrils demonstrated statistically significant changes at 75, 150, and 250 mg (vs placebo).

The change in PD with amiloride for the three analysis methods is shown in Figure S2 in File S1. Dose-dependent effects were observed for the average of both nostrils and the most-polarized nostril at each visit, but results with this parameter were less clear and consistent than with the basal PD and Ringer's PD estimates of ENaC activity. The percent change in amiloride was particularly difficult to interpret with no clear dose effects seen (Figure S3 in File S1).

### Changes in delta NPD

**Figure 4:**
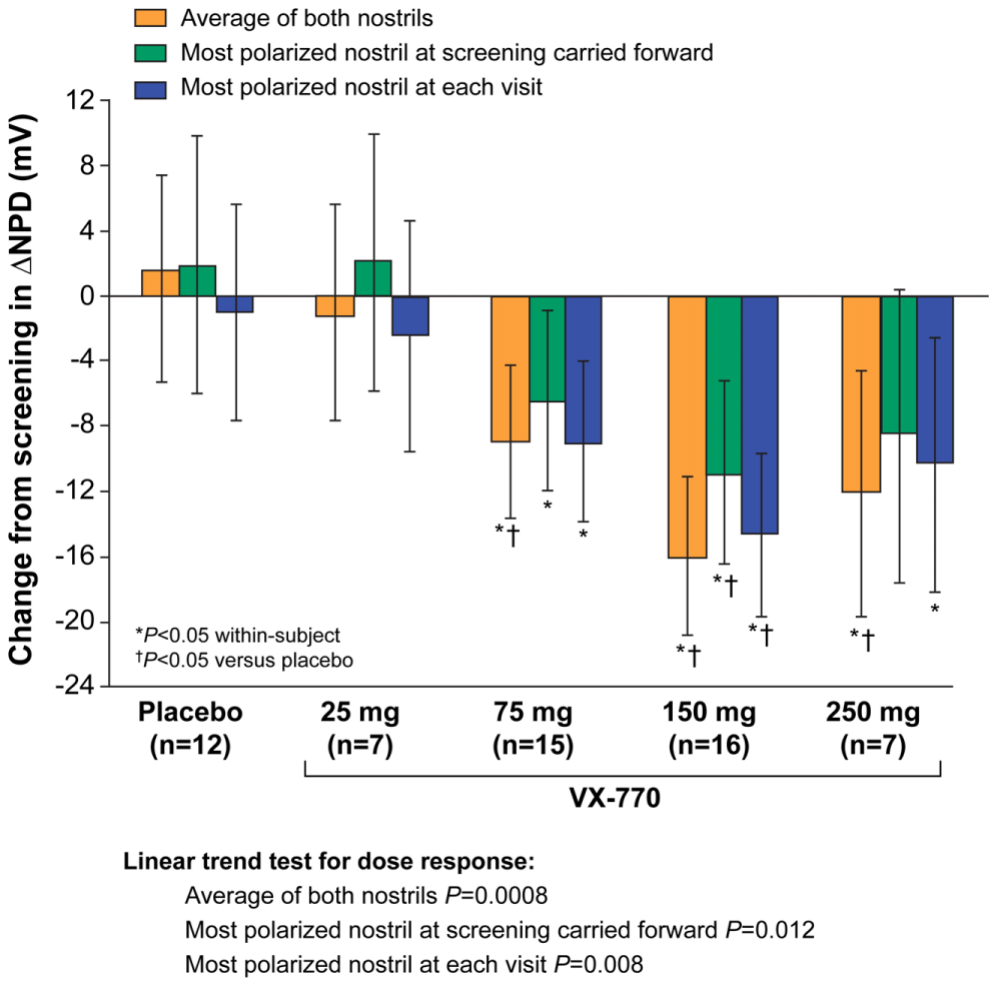
Change from screening to Day 14 in delta NPD, Parts 1 and 2 combined. Data are shown for the analysis of the average of both nostrils, the most-polarized nostril at screening carried forward, and the most-polarized nostril at each visit. The dose-dependent linear trends were statistically significant for all three analyses (*P*<0.02).

The change in delta NPD has not been used commonly in clinical trials of CFTR modulators or in gene replacement studies, but two recent cross-sectional studies provided evidence that this measurement correlates with disease phenotype [8], [27]. Similar to sweat Cl^−^, delta NPD incorporates both Na^+^ and Cl^−^ transport (ENaC and CFTR activity) into a single measurement, potentially reflecting the balance of Na^+^ absorption and Cl^−^ secretion in the epithelium. Changes in the delta NPD parameter are shown in Figure 4; ivacaftor produced dose-dependent improvements based on all three analysis methods (*P*<0.02), with diminished effects at the 250-mg dose as was observed with sweat Cl^−^ testing in this population. None of the NPD measures (Na^+^, Cl^−^, or the delta NPD) correlated with changes in lung function (FEV_1_, FVC, or FEF_25%–75%_).

### Sample-Size Estimates

These data provide an evidence-basis for the design of future trials using NPD. We derived sample-size data from NPD effects seen with the 150-mg ivacaftor dose, because it included the largest number of study subjects and demonstrated evidence of bioactivity based on Cl^−^, Na^+^, and delta NPD measurements (Tables 2, 3, 4). For each table, we illustrate sample sizes required to provide 80% and 90% power for the average of both nostrils and the most-polarized nostril at each visit; the most-polarized nostril at screening carried forward method was inferior in each case and therefore not included. A large number of subjects are required to demonstrate a statistically significant change in CFTR activity (zero Cl^−^ plus isoproterenol) between groups, regardless of the analysis method (Table 2A). For within-subject analysis, sample sizes are reasonable for Phase II trial designs in CF (Table 2B). As few as 10 subjects per group would be sufficient to detect a 5 mV treatment effect (the postulated effect size to indicate a meaningful difference in clinical phenotype prior to this trial and a criterion previously used to define responders in CF modulator studies [8], [28]).

**Table 2:** Sample-size estimates based on zero Cl^−^ plus isoproterenol (CFTR) response*.

Treatment Effect (mV)	Total Number of Subjects					
	90% Power			80% Power		
	Average of Both Nostrils	Most-polarized nostril at Each Visit	Most-polarized nostril at Screening Carried Forward	Average of Both Nostrils	Most-polarized nostril at Each Visit	Most-polarized nostril at Screening Carried Forward
**A. Comparison with placebo**						
−1	854	676	854	638	506	638
−2	216	172	216	162	128	162
−3	98	78	98	74	58	74
−4	56	46	56	42	34	42
−5	38	30	38	28	24	28
**B. Within-group comparison** **						
−1	215	188	215	161	149	161
−2	56	49	56	42	37	42
−3	26	23	26	20	18	20
−4	16	14	16	12	11	12
−5	11	10	11	9	8	9

* Parts 1 and 2 combined analysis at Day 14.

** Assume a single-arm study.

**Table 3:** Sample-size estimates based on average basal PD (ENaC)*.

Treatment Effect (mV)	Total Number of Subjects					
	90% Power			80% Power		
	Average of Both Nostrils	Most-polarized nostril at Each Visit	Most-polarized nostril at Screening Carried Forward	Average of Both Nostrils	Most-polarized nostril at Each Visit	Most-polarized nostril at Screening Carried Forward
**A. Comparison with placebo**						
−5	154	140	226	116	104	170
−10	40	38	58	32	28	44
−15	20	18	28	16	14	22
−20	12	12	18	10	10	14
−25	10	8	12	8	8	10
**B. Within-group comparison** **						
−5	40	37	58	31	28	44
−10	12	11	16	10	9	13
−15	7	7	9	6	6	7
−20	5	5	6	5	4	5
−25	4	4	5	4	4	4

* Parts 1 and 2 combined analysis at Day 14.

** Assume a single-arm study.

**Table 4:** Sample-size estimates based on delta NPD*.

Treatment Effect (mV)	Total Number of Subjects					
	90% Power			80% Power		
	Average of Both Nostrils	Most-polarized nostril at Each Visit	Most-polarized nostril at Screening Carried Forward	Average of Both Nostrils	Most-polarized nostril at Each Visit	Most-polarized nostril at Screening Carried Forward
**A. Comparison with placebo**						
−5	288	206	226	216	154	170
−10	74	54	58	56	42	44
−15	34	26	28	26	20	22
−20	20	16	18	16	12	14
−25	14	12	12	12	10	10
**B. Within-group comparison** **						
−5	73	53	58	56	40	44
−10	26	15	16	16	12	13
−15	11	8	9	9	7	7
−20	7	6	6	8	5	5
−25	6	5	5	5	4	4

* Parts 1 and 2 combined analysis at Day 14.

** Assume a single-arm study.

Sample-size estimates also were examined for ENaC activity based on average basal PD, maximal basal PD, and Ringer's PD (Figures 2 and 3, Figure S2 in File S1). For the change in average basal PD, sample sizes for the average of both nostrils and the most-polarized nostril at each visit are shown with 80% and 90% power for between-group (Table 3A) and within-group (Table 3B) assessment methods. For the average basal PD, the average of both nostrils and the most-polarized nostril at each visit demonstrated the smallest sample sizes required to show a significant within-group effect (Table 3). Within-group and between-group variances for maximal basal PD and Ringer's PD were identical, thus the sample size estimates from these parameters were the same and are shown together in Table S2 in File S1.

Sample-size estimates for the delta NPD (both ENaC and CFTR activity) using the three analyses are shown in Table 4 and were similar to that observed for Cl^−^ transport. To detect a change of −15 mV (approximating the mean effect magnitude in subjects with CF treated with 150 mg of ivacaftor), seven to nine subjects are required for within-group comparisons with 80% power. Together, these results suggest that the greatest sensitivity to detect a change in PD in patients with CF exposed to a CFTR modulator is achieved by directly measuring CFTR activity, or jointly estimating CFTR and ENaC activity by the delta NPD.

### Consistency of NPD Parameters in Placebo-Treated Subjects Across Modulator Trials

Based on the results and experience in the Phase II ivacaftor study, the NPD protocol was modified for the subsequent placebo-controlled, Phase II lumacaftor trial, including the institution of electronic data capture and use of an agar probe electrode as previously described [26]. The two study groups were generally similar, enrolling only adult subjects with two known *CFTR* mutations and FEV_1_>40% predicted [22], [26]. Using a combined placebo dataset derived from both trials (n = 25 subjects, average of both nostrils), the mean difference (± standard deviation [SD]) from screening to Day 14 in PD parameters were consistent with the ivacaftor-only trial, including (1) zero Cl^−^ plus isoproterenol (CFTR activity) = 0.21 mV (±4.15 SD); (2) average basal PD (ENaC activity) = 1.75 mV (±9.80 SD); and (3) delta NPD (CFTR and ENaC activity) = 0.15 mV (±9.75 SD).

## Discussion

The NPD is a CFTR biomarker capable of isolating CFTR activity, segregates patients based on CFTR function, is a recognized diagnostic test for CF, and has been a critical endpoint in early phase clinical trials of CFTR modulators [8]–[22]. In the current report, we provide a comprehensive analysis of NPD endpoints from a successful multicenter CFTR modulator clinical trial comprising 39 subjects. This was the largest placebo-controlled study evaluating an efficacious CFTR modulator completed to date, and the first multicenter trial to use centralized solutions and other standardization procedures to improve NPD testing consistency. The Phase III studies of ivacaftor in patients did not include the NPD, and the Phase II study of VX-809 monotherapy in CF patients with two F508del CFTR mutations did not demonstrate improvement in the NPD or in clinical efficacy measures [29], [30]. The positive and consistent findings in both CFTR biomarkers and clinical outcome measures from the Phase II study of ivacaftor in G551D-CF patients provided a unique opportunity to determine the optimal measures for quantifying CFTR activity and inform future decisions in NPD analysis. The average of both nostrils and the most-polarized nostril at each visit achieved robust statistical significance for most parameters, including changes in Cl^−^ transport, change in Na^+^ transport (average basal PD, maximal basal PD, Ringer's PD, and change in PD with amiloride), and a combined measure of ion transport (delta NPD). In contrast, analysis based on the most-polarized nostril at screening carried forward was less robust, with greater variability and smaller treatment effects observed (particularly for the parameters measuring ENaC activity; see Figures 2, 3, 4 and Figures S2, S3 in File S1). These results support the conclusion that performance of NPD should include both nostrils in clinical trials.

NPD has historically been a challenging assay requiring rigorous conduct to ensure consistent and valid results. Despite significant expansion of the number of participating centers and operators in the trial described here, the within-subject variability of CFTR activity [change in zero Cl^−^ plus isoproterenol (SD = ±2.8 mV for placebo-treated subjects using the average of both nostrils and ±2.7 mV for the most-polarized nostril at each visit)] was lower than in previous multicenter trials that used a smaller number of experienced centers and topical agents (±5.1 mV) [24] or systemic drugs (±5.2 mV) [31]. This also compares favorably with reproducibility of this measurement from single-center studies [12], [20]. The rigorous standardization of the NPD procedure conducted prior to the start of this trial may have contributed to the consistent within-subject variance, despite the addition of several new centers to the NPD network. Included among these were the use of a standard operating procedure, qualification procedures for NPD operators to demonstrate proficiency (including hands-on supervision and training), use of centralized NPD solutions (with accompanying quality assurance), and centralized NPD interpretation (including a strict blinding process). Attributing the consistent within-subject variance to rigorous standardization of the procedure is further supported by the fact that >85% of tracings were deemed acceptable for review (based on tracing stability at solution changes and absence of tracing artifact). This was confirmed in the Phase II lumacaftor study in which the placebo cohort showed consistency across key NPD parameters despite the addition of seven study sites.

The Na^+^ transport (ENaC) parameters that best detected changes following ivacaftor treatment included average basal PD, maximal basal PD, and Ringer's PD, with no single Na^+^ transport parameter clearly outperforming the other two (Figures 2, 3, and Figure S2 in File S1). In contrast, the absolute change and percent change in amiloride response were less consistent and generally failed to demonstrate clear treatment effects (Figures S3, S4 in File S1). While this may be due to the fact that the effects on ENaC activity with ivacaftor are indirect, we speculate that the parameters measuring ENaC activity, particularly changes in PD with amiloride, may be relatively dependent upon catheter placement since the resting PD can vary greatly when separated by small distances within the inferior meatus. The CFTR activity measurement, in contrast, may be less dependent upon placement since the prominent effects of ENaC are eliminated by amiloride perfusion.

We observed discordant findings when examining ENaC and CFTR activity in the highest ivacaftor dose cohort (Figures 1, 2, 3, Figures S2, S3, S4 in File S1), with continued improvements in CFTR activity accompanied by no further improvements in ENaC activity. The delta NPD dose-response curve (Figure 4) was qualitatively similar to that previously reported for sweat Cl^−^, which also exhibited the greatest bioactivity in the 150-mg dose group. The results support the notion that, like sweat Cl^−^, delta NPD provides an aggregate measure of CFTR and ENaC activity [22].

Power analyses indicate that sample sizes needed to detect a −3 mV change in CFTR activity (zero Cl^−^ plus isoproterenol; n = 20 for average of both nostrils, n = 18 for the most-polarized nostril at each visit) based on within-group analysis and 80% power should be sufficient to detect modest changes in ENaC activty (+10 mV for the average basal PD, average of both nostrils or the most-polarized nostril at each visit; Table 3). Although this example would be underpowered to detect differences between treatment group and placebo, we recommend including a placebo arm to help preserve the integrity of the analysis by a blinded interpreter.

The results of our study indicate that both nostrils should be included when performing the NPD as part of interventional trials, since this allows measurement of the average PD from both nostrils and the most-polarized nostril at each visit. Measurements dependent on both nostrils were superior to using a single nostril (i.e., the most-polarized nostril at screening carried forward), an inference that previously has been suggested [32]. Since the average of both nostrils tended to be less variable compared with the other methods assessing CFTR and ENaC activity, we recommend this as the principle method used for NPD testing. The current dataset provides guidance for NPD use in future modulator trials and confirms the value of rigorous standardization on assay performance.

## Supporting Information

File S1 **Includes Methods S1, Figure Legends S1–S4, Figures S1, S2, S3, S4, Tables S1 and S2, and References S1 sections.**
(DOC)
