# Supplementary material for: Optimizing Nasal Potential Difference Analysis for CFTR Modulator Development: Assessment of Ivacaftor in CF Subjects with the G551D-CFTR Mutation
Source: PLoS One. 2013 Jul 26;8(7):e66955. doi: 10.1371/journal.pone.0066955 (PMC3724869; doi:10.1371/journal.pone.0066955)
Supplement: File S1 — Includes Methods S1, Figure Legends S1–S4, Figures S1, S2, S3, S4, Tables S1 and S2, and References S1 sections. (DOC) [file pone.0066955.s001.doc]

**Optimizing Nasal Potential Difference Analysis for CFTR Modulator Development: Assessment of Ivacaftor in CF Subjects with the *G551D-CFTR* Mutation**

Steven M. Rowe^1^, Bo Liu^1^, Aubrey Hill^1^, Heather Hathorne^1^, Morty Cohen^2^, John Beamer^2^, Frank Accurso^3^, Qunming Dong^4^, Claudia L. Ordoñez^5^, Anne Stone^4^, Eric Olson^4^, and *John P. Clancy^6^; for the VX06-770-101 study group

**Affiliations:**

^1^University of Alabama at Birmingham, Birmingham, Alabama, United States of America

^2^Formerly Seattle Children’s Hospital, Seattle, Washington, United States of America; currently private consulting

^3^University of Colorado Denver, Aurora, Colorado, United States of America

^4^Vertex Pharmaceuticals Incorporated, Cambridge, Massachusetts, United States of America

^5^Formerly Vertex Pharmaceuticals Incorporated, Cambridge, Massachusetts, United States of America; currently employed by Biogen

^6^Cincinnati Children’s Hospital Medical Center and the University of Cincinnati, Cincinnati, Ohio, United States of America

**SUPPORTING INFORMATION**

**METHODS S1**

*Nasal Potential Difference (NPD) Performance in Human Subjects*

The NPD assessment in this study was based on that originally reported by Knowles and colleagues , with several modifications for use in this multicenter trial. First, all solutions were centrally produced (University of California, San Francisco Research Pharmacy, San Francisco, CA) and distributed to sites by the Cystic Fibrosis Foundation Therapeutic Development Network Coordinating Center (CFF-TDN CC) in Seattle, WA. Solution content was as described by Knowles, and sterility, electrolyte, and amiloride stability testing were conducted by the CFF-TDN CC. Isoproterenol and ATP were added to the appropriate NPD solutions ≤ 2 hours prior to the procedure, and all solutions were warmed to 32°-37°C. All NPD operators underwent a qualification process prior to initiating the study. The qualification process included submission of acceptable NPD measurements in five subjects with CF and five non-CF subjects as reviewed by the TDN NPD Interpretative Center at the University of North Carolina at Chapel Hill (prior to April 2006) or the Center for CFTR Detection at the University of Alabama at Birmingham (after April 2006). In addition, NPD measurements from one subject with CF and one non-CF subject were reviewed by the Center for CFTR Detection within 12 months of the study start (typically within one month of study-subject enrollment in the ivacaftor trial). The Center for CFTR Detection observed the performance of an NPD procedure by operators from each site, either locally or at NPD workshops. All NPDs were reviewed by a single NPD interpreter. For NPDs in the clinical trial, the NPD interpreter was blinded from identifying information (e.g., subject, site, date, visit number, etc.).

*NPD Analysis*

Examples of NPD recordings are shown in Supplemental Figure 1. The primary NPD endpoint parameter was the change in PD following zero Cl^–^ plus isoproterenol perfusion. Secondary parameters included the average basal PD (the mean of five locations within the inferior meatus), the maximal (most polarized) basal PD, Ringer’s PD (change in PD following one minute of Ringer’s solution perfusion), and the change with amiloride PD (change in PD following three minutes of amiloride perfusion), each a measure of sodium conductance. The ΔNPD (difference between PD at completion of zero Cl^–^ plus isoproterenol perfusion and PD with Ringer’s perfusion) was calculated as an aggregate measure of Na^+^ and Cl^–^ transport.

The primary analysis method for each NPD parameter was the average of both nostrils. Alternative NPD analysis methods also evaluated for each parameter included the most-polarized nostril at screening carried forward and the most-polarized nostril at each visit.

*Statistical Analysis*

Analyses were performed on all randomized subjects who received at least one dose of study drug. For pooled analyses of data from Parts 1 and 2 at Day 14, a mixed model was used and the least-squares-mean change from baseline was obtained from the model. Statistical analyses were performed using SAS Version 9.1 (SAS Institute Inc., Cary, NC). The power analysis is based on a two-sample t-test for comparisons with placebo (assuming a 1:1 randomization ratio) and a one-sample t-test for within-group comparisons (assuming a single treatment arm only).

*Reagents*

The details of the NPD reagents used are included in the CFF-TDN NPD Standard Operating Procedures I (2008), available upon request from the TDN Coordinating Center (http://www.cff.org/research/TDN/). Analysis of electrolyte content was performed by Galbraith Laboratories, Inc. (Knoxville, TN) and amiloride content was measured by using reverse-phase high-performance liquid chromatography and mass spectrometry (also by Galbraith Laboratories, Inc.)

**FIGURE LEGENDS**

**Figure S1.** **Examples of NPD recordings.** *Left*: Basal PD measurements at discrete distances under the inferior turbinate in a non-CF subject (AT = anterior tip of the inferior turbinate; 3 cm, 2 cm, 1.5 cm, 1 cm, and 0.5 cm). *Right*: NPD tracings in a subject with CF (red) and a non-CF subject (black). The initiation of each perfusion solution is designated.

**Figure S2.** **Change from screening to Day 14 in Ringer’s PD, Parts 1 and 2 combined.** Data are shown for the analysis of the average of both nostrils, the most-polarized nostril at screening carried forward, and the most-polarized nostril at each visit. Statistically significant dose-dependent linear trends were demonstrated for the average of both nostrils (*P* = 0.003) and the most-polarized nostril at screening carried forward (*P* = 0.03).

**Figure S3.** **Change from screening to Day 14 in amiloride response, Parts 1 and 2 combined.** Data are shown for the average of both nostrils, the most-polarized nostril at screening carried forward, and the most-polarized nostril at each visit. Statistically significant dose-dependent linear trends were demonstrated for the average of both nostrils (*P* = 0.02).

**Figure S4. Percent change from screening to Day 14 in amiloride response, Parts 1 and 2 combined.** Data are shown for the analysis of the average of both nostrils, the most-polarized nostril at screening carried forward, and the most-polarized nostril at each visit. There were no dose-dependent linear trends demonstrated for the different measurements.

**Figure S1.** **NPD measurements analyzed.**

**
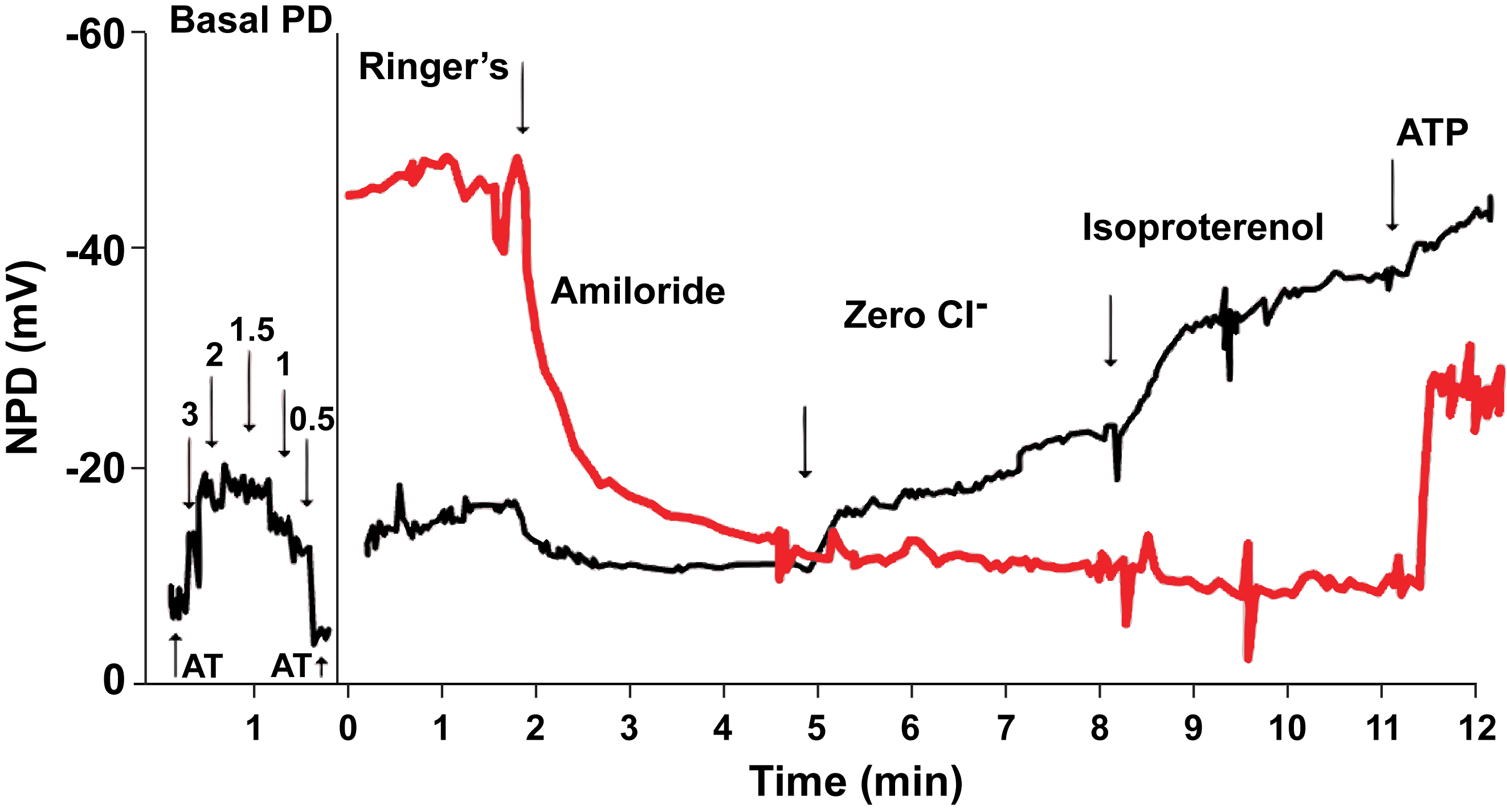
**

**Figure S2.** **Change from screening to Day 14 in Ringer’s PD, Parts 1 and 2 combined.**


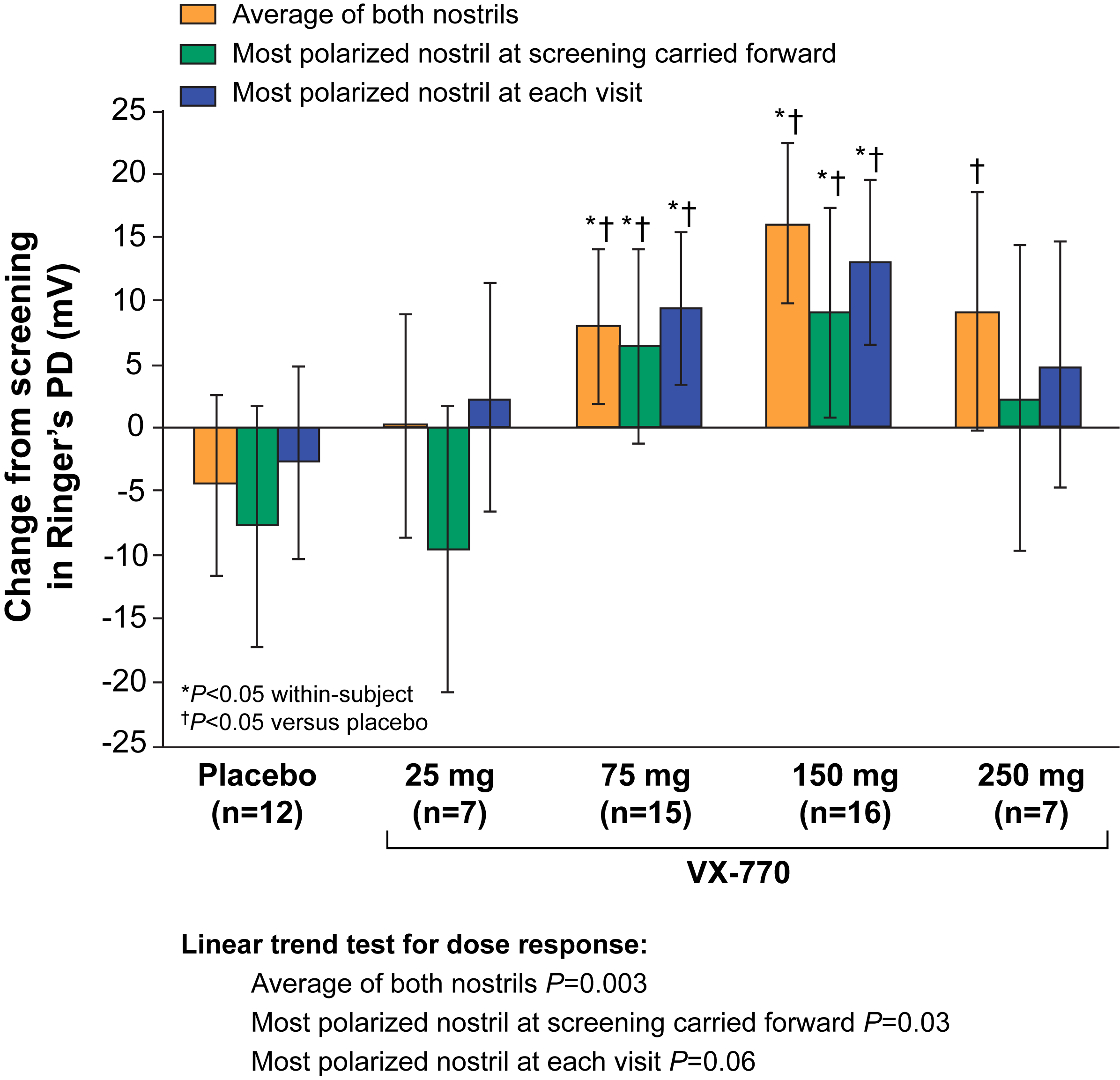


**Figure S3.** **Change from screening to Day 14 in amiloride response, Parts 1 and 2 combined.**

**
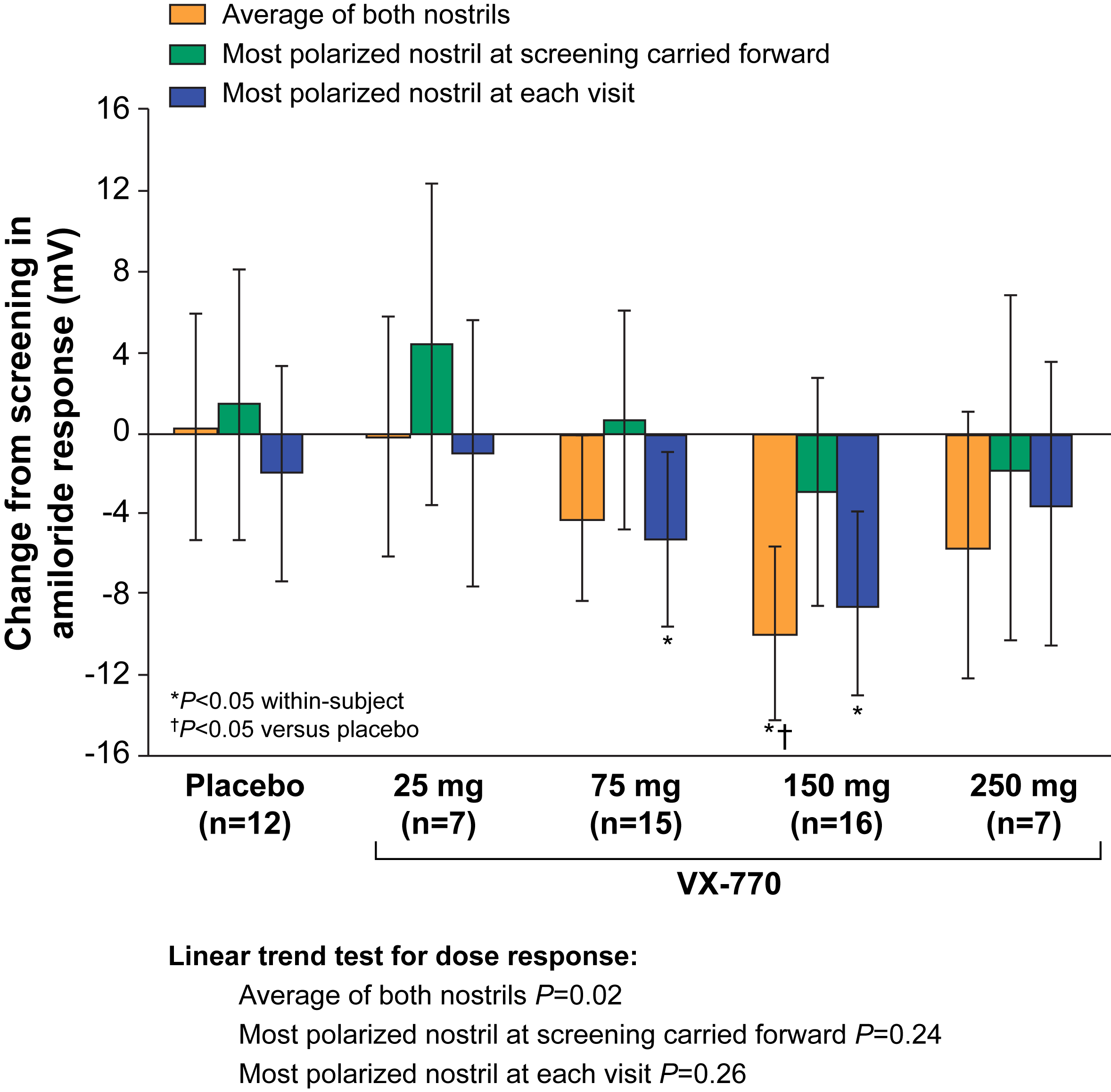
**

**Figure S4. Percent change in amiloride response from screening to Day 14, Parts 1 and 2 combined.**


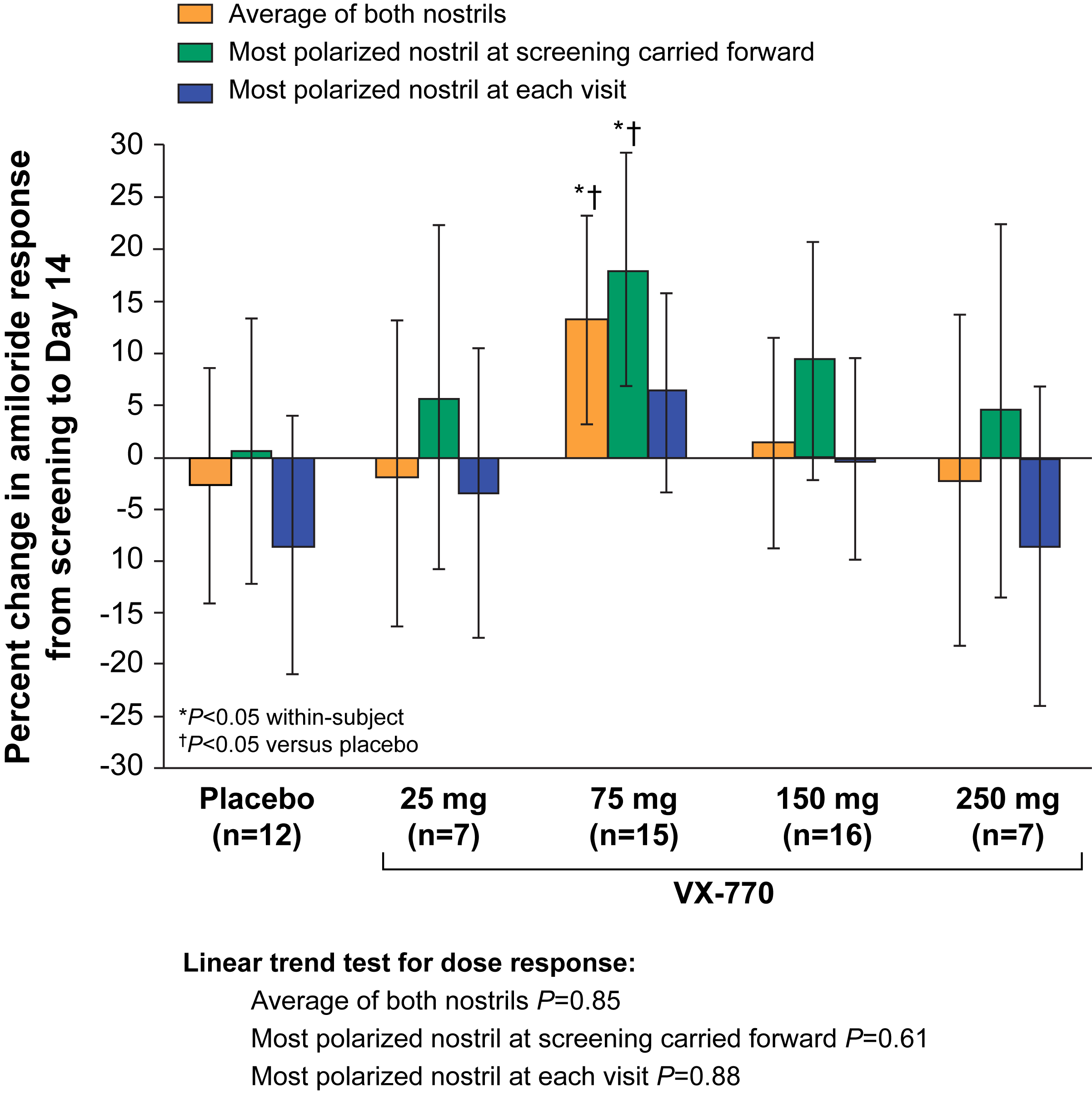


**Table S1.** Electrolyte and amiloride concentrations and pH of NPD solutions over time

|  | **Time (months)**  **Measure ± SD** | | | | | | |
| --- | --- | --- | --- | --- | --- | --- | --- |
|  | **Target** | **Screening** | **2** | **4** | **6** | **8** | **12** |
| **Electrolyte** | | | | | | | |
| Sodium, mEq/L (n=5) | 148 | 153.8±8.5 |  |  |  | 155.5±3.5 | 166.7±3.1 |
| Chloride, mEq/L (n=3) | 156 | 159.0±3.4 |  |  |  | 149.0±0 | 149.0±0 |
| Calcium, mEq/L (n=5) | 4.5 | 4.8±0.1 |  |  |  | 4.7±0.1 | 4.6±0.1 |
| **Amiloride,** mg/L (n=7) | 30 | 33.1±2.9 | 31.3±0.4 | 30.0±0.1 | 31.8±2.9 | 29.6±2.1 | 29.7±1.2 |
| **pH** (n=5) | 7.1-7.6 | 7.37±0.17 |  |  |  | 7.38±0.07 | 7.35±0.07 |

SD, standard deviation

**Table S2. Sample-size estimates based on maximum basal PD and Ringer’s PD***

| **Treatment Effect (mV**) | **Number of Subjects** | | | | | |
| --- | --- | --- | --- | --- | --- | --- |
|  | **90% Power** | | | **80% Power** | | |
|  | Average of Both Nostrils | Most-polarized nostril at Each Visit | Most-polarized nostril at Screening Carried Forward | Average of Both Nostrils | Most-polarized nostril at Each Visit | Most-polarized nostril at Screening Carried Forward |
| 1. **Comparison with placebo** | | | | | | |
| -5 | 310 | 288 | 288 | 232 | 216 | 216 |
| -10 | 80 | 74 | 74 | 60 | 56 | 56 |
| -15 | 38 | 34 | 34 | 28 | 26 | 26 |
| -20 | 22 | 20 | 20 | 18 | 16 | 16 |
| -25 | 16 | 14 | 14 | 12 | 12 | 12 |
| 1. **Within-group comparison**** | | | | | | |
|  | 79 | 73 | 73 | 60 | 56 | 56 |
| -10 | 22 | 26 | -5 | 17 | 16 | 16 |
| -15 | 11 | 11 | 11 | 9 | 9 | 9 |
| -20 | 7 | 7 | 7 | 6 | 8 | 8 |
| -25 | 6 | 6 | 6 | 5 | 5 | 5 |

*Parts 1 and 2 combined analysis at Day 14 **Assume a single-arm study

**REFERENCES S1**

1. Knowles MR, Paradiso AM, Boucher RC (1995) In vivo nasal potential difference: techniques and protocols for assessing efficacy of gene transfer in cystic fibrosis. Hum Gene Ther 6: 445-455.
